# Lignin-degrading enzymes from a pathogenic canker-rot fungus *Inonotus obliquus* strain IO-B2

**DOI:** 10.1186/s13568-023-01566-3

**Published:** 2023-06-11

**Authors:** Retno Agnestisia, Tomohiro Suzuki, Akiko Ono, Luna Nakamura, Ikumi Nezu, Yuki Tanaka, Haruna Aiso, Futoshi Ishiguri, Shinso Yokota

**Affiliations:** 1 United Graduate School of Agricultural Science, Tokyo University of Agriculture and Technology, Fuchu, Tokyo, 183-8509 Japan; 2 School of Agriculture, Utsunomiya University, Utsunomiya, Tochigi 321-8505 Japan; 3 Faculty of Mathematics and Natural Sciences, Universitas Palangka Raya, Palangka Raya, 73111 Indonesia; 4 Center for Bioscience Research and Education, Utsunomiya University, Utsunomiya, Tochigi 321-8505 Japan; 5 Faculty of Agricultural Production and Management, Shizuoka Professional University of Agriculture, Iwata, Shizuoka, 438-0803 Japan

**Keywords:** *Inonotus obliquus*, Pathogenic canker-rot fungus, Lignin-degrading enzymes, Manganese peroxidase, Genome sequence

## Abstract

*Inonotus obliquus* is a pathogenic fungus found in living trees and has been widely used as a traditional medicine for cancer therapy. Although lignocellulose-degrading enzymes are involved in the early stages of host infection, the parasitic life cycle of this fungus has not been fully understood. In this study, we aimed to investigate the activities of laccase (Lac), manganese peroxidase (MnP), and lignin peroxidase (LiP) from *I. obliquus* cultivated in Kirk’s medium. The fungus was subjected to genome sequencing, and genes related to wood degradation were identified. The draft genome sequence of this fungus comprised 21,203 predicted protein-coding genes, of which 134 were estimated to be related to wood degradation. Among these, 47 genes associated with lignin degradation were found to have the highest number of *mnp* genes. Furthermore, we cloned the cDNA encoding a putative MnP, referred to as IoMnP1, and characterized its molecular structure. The results show that IoMnP1 has catalytic properties analogous to MnP. Phylogenetic analysis also confirmed that IoMnP1 was closely related to the MnPs from *Pyrrhoderma noxium*, *Fomitiporia mediterranea*, and *Sanghuangporus baumii*, which belong to the same family of Hymenochaetaceae. From the above results, we suggest that IoMnP1 is a member of MnPs.

**Supplementary Information:**

The online version contains supplementary material available at 10.1186/s13568-023-01566-3.

## Introduction

**Fig. 1:**
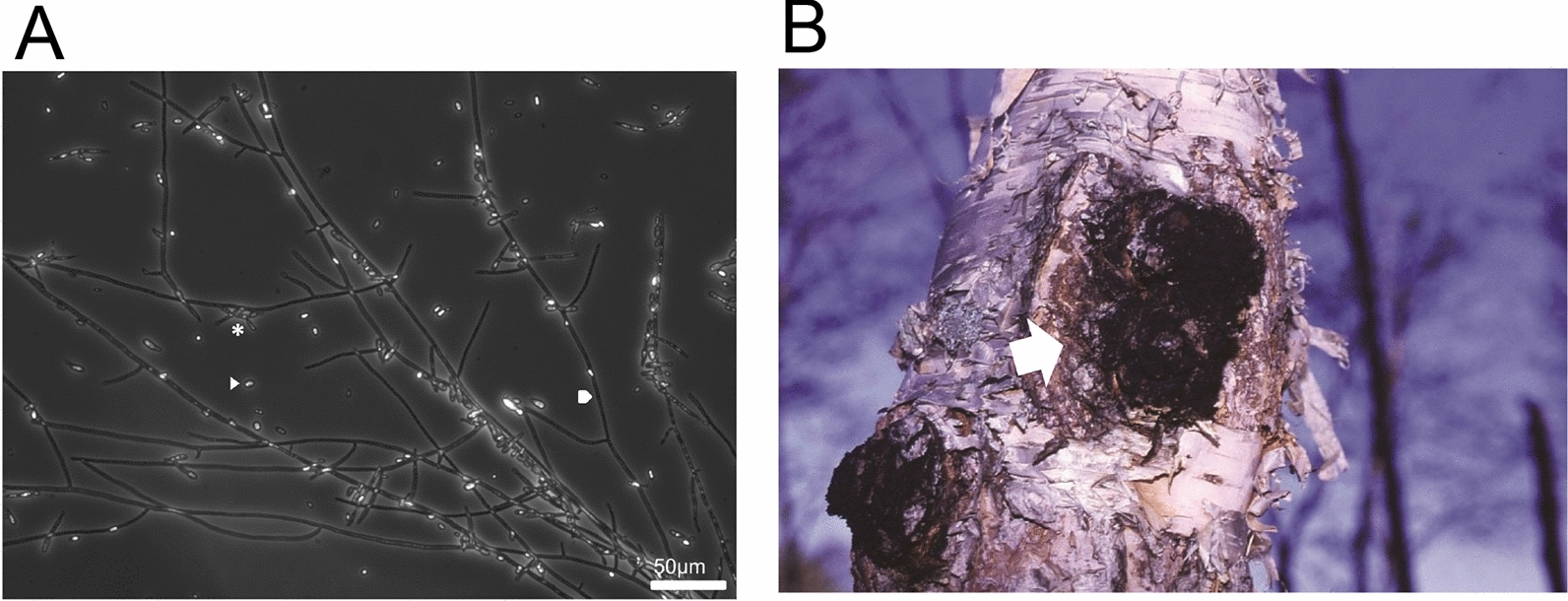
White-rot fungus *I. obliquus*. **A** Micrograph of *I. obliquus* mycelia by phase-contrast microscopy. Pentagon and arrowhead indicate mycelium and conidium, respectively. In this case, *I. obliquus* mycelia are still germinating (asterisk). Scale bar, 50 μm. **B** Photograph of *I. obliquus* sclerotium (white arrow) on a Japanese birch tree. The photograph was taken by Shinso Yokota

*Inonotus obliquus* (Fr.) Pilát, commonly known as “Chaga” in Russia and “Kabanoanatake “ in Japan, is a member of the white-rot fungi belonging to family *Hymenochaetaceae* (Fig. 1A). Unlike other white-rot fungi, this fungus parasitizes living trees by forming sclerotia or canker-like bodies called “Chaga “ (Fig. 1B). Therefore, *I. obliquus* is often referred to as a pathogenic canker-rot fungus (Blanchette 1982; Lee et al. 2008). Several researchers have reported that the parasitic life cycle of *I. obliquus* in living trees displays unique features (Shashkina et al. 2006; Lee et al. 2008). The basidiospores of this fungus disperse in the air and fall onto damaged areas of living birch trees, grow into the wood, and form a mycelium. The hyphae of the mycelium then penetrate the tree through wounds, destroy it, and form sclerotia (thick-walled black mycelia) after 10–15 years of parasitism (Shashkina et al. 2006; Lee et al. 2008).

Several studies have shown that *I. obliquus* can degrade cellulose, hemicellulose, and lignin in the biomass of non-woody plants such as corn straw (Chen et al. 2011), rice straw, sugarcane bagasse (Xu et al. 2014), wheat straw (Xu et al. 2019), and *Eucommia ulmoides* leaves (Qian et al. 2020). Xu et al. (2017) examined the enzymatic activities of *I. obliquus* in degrading cellulose, hemicellulose, and lignin in wheat straw, rice straw, and corn stover (Xu et al. 2017). They determined the enzyme activities of β-glucosidase, xylanase, and manganese peroxidase in *I. obliquus* (Xu et al. 2017). Xu et al. (2018) also found that *I. obliquus* was able to produce highly efficient activity of cellulose-degrading enzymes for CMCase (27.15 IU/g), FPase (3.16 IU/g) and β-glucosidase (2.53 IU/g) using wheat bran under solid state fermentation. Zang et al. (2021) discovered that the lignin-degrading enzymes in *I. obliquus* could be used to produce animal feed from wheat straw. These enzymes were lignin peroxidase, manganese peroxidase, and laccase with enzyme activities of 1729, 610 and 98 IU/g, respectively. However, further study is required to fully understand the enzymatic systems of *I. obliquus* involved in degrading the chemical components of plant cell walls, especially in woody plants.

Lignin is a chemical component of wood cell walls that is most efficiently degraded by white-rot fungi in nature (Chi et al. 2007). Therefore, it is important to investigate the lignin-degrading enzyme activity of *I. obliquus*. Lignin-degrading enzymes can generally be classified into lignin-modifying enzymes and lignin-degrading auxiliary enzymes (Janusz et al. 2017). Lignin-modifying enzymes are typically oxidative enzymes classified into two types: (1) Laccase (Lac; EC 1.10.3.2), a copper-containing phenol oxidase that can directly oxidize the phenolic units of lignin using molecular oxygen (O_2_) as an oxidizing agent (Manavalan et al. 2015; Janusz et al. 2017) and (2) Heme-containing peroxidases that can oxidize the phenolic or non-phenolic units of lignin using hydrogen peroxide (H_2_O_2_) as an oxidizing agent (Manavalan et al. 2015). In contrast, lignin-degrading auxiliary enzymes provide H_2_O_2_ for heme-containing peroxidases to complete the lignin degradation process (Janusz et al. 2017).

Burton (2003) reported that heme-containing peroxidases are biocatalysts that are widely used for various applications compared to laccases because of their high redox potential. Therefore, heme-containing peroxidases are highly attractive enzymes to investigate. Heme-containing peroxidases can be classified into manganese peroxidase (MnP; EC 1.11.1.13), lignin peroxidase (LiP; EC 1.11.1.14), and versatile peroxidase (VP; EC 1.10.3.2) (Janusz et al. 2017). MnP is characterized by a Mn-binding site (Sundaramoorthy et al. 1994). At the Mn-binding site, Mn^2+^ is oxidized to Mn^3+^, which is then chelated by organic acids. The resultant Mn^3+^–organic acid complex acts as a diffusible redox mediator to oxidize the phenolic units of lignin (Manavalan et al. 2015). Unlike MnP, LiP is characterized by an invariant tryptophan (Trp) residue located on the enzyme surface (Choinowski et al. 1999). This residue is involved in the long-range electron transfer (LRET) pathway to oxidize the non-phenolic units of lignin (Choinowski et al. 1999). These structural features of both MnP and LiP are present in VP (Camarero et al. 1999; Ruiz-Dueñas et al. 1999).

In recent years, genome sequencing technology and bioinformatics tools have been the most efficient methods for rapidly providing whole-genome information at the molecular level for a fungus. The genomes of many white-rot fungi have been sequenced, including *Heterobasidion irregulare* (Olson et al. 2012), *Ganoderma lucidum* (Liu et al. 2012), *Trametes versicolor* (Floudas et al. 2012), *Phanerochaete chrysosporium* (Ohm et al. 2014), *Phanerochaete carnosa* (Ohm et al. 2014), *Lentinula edodes* (Chen et al. 2016) and *Pleurotus eryngii* (Li et al. 2018). In addition, different types of enzymes-encoding genes that involved in lignin degradation have been characterized (Janusz et al. 2013). To date, cDNA cloning of *lip*, *mnp*, and *vp* has been conducted in several species, including Polyporales, Agaricales, and Corticiales Basidiomycetes (Camarero et al. 1999; Ruiz-Dueñas et al. 1999, 2009; Martinez 2002; Moreira et al. 2005; Mohorčič et al. 2009; Janusz et al. 2013; Schüttmann et al. 2014).

In the present study, we investigated the activity of lignin-degrading enzymes in *I. obliquus*. We also conducted genome sequencing and identified the genes related to wood degradation. This information is expected to provide new insights into the enzymatic systems of *I. obliquus* that are involved in degrading wood chemical components, especially lignin. From these results, we cloned one of the cDNAs encoding putative MnP and characterized its structural features. Finally, we performed phylogenetic analysis to investigate the evolutionary relationships of this enzyme with other Basidiomycete peroxidases.

## Methods

### Fungal strain

*Inonotus obliquus* strain IO-B2 (NBRC 113408) from the Forest Resource Biology, Forest Resource Science, Division of Environmental Resources, Graduate School of Agriculture, Hokkaido University, Japan, was used in this study. This fungus was pre-cultured on 39 g/L (w/v) potato-dextrose-agar (PDA; Becton, Dickinson and Company, Sparks, MD, USA) medium in a 9-cm diameter Petri dish at 25 ± 2 °C in the dark.

### Enzyme activity assays

The actively growing *I*. *obliquus* mycelia were obtained from a PDA medium and inoculated into 300 mL-Erlenmeyer flasks containing 20 mL of Kirk medium (Kirk et al. 1986) with the following composition (/L medium):100 mL Basal III medium (2 g KH_2_PO_4_, 500 mg MgSO_4_•7H_2_O, 5 mg thiamine•HCl, 150 mg CaCl_2_•2H_2_O, and 70 mL mineral solution (3 g MgSO_4_•7H_2_O, 56.2 mg ZnSO_4_•7H_2_O, 10 mg H_3_BO_3_, 8.5 mg Na_2_MoO_4_•2H_2_O, 1.4 g nitrilotriacetate, 350 mg MnSO_4_•5H_2_O, 100 mg FeSO_4_•7H_2_O, 5.4 mg AlK(SO_4_)_2_•12H_2_O, 10 mg CuSO_4_•5H_2_O, 55.2 mg CoSO_4_•7H_2_O, 1 g NaCl, and 82 mg CaCl_2_/1 L H_2_O)/1 L H_2_O), 50 mL 120 mM ammonium tartrate, 100 mL 0.1 M *t*-acconitate buffer (pH 4.3), 10 mL 20% D-glucose aqueous solution, and 40 mL 2.5% Tween 80 (Kirk et al. 1986). The medium was inoculated with 10 agar plugs (5 mm diameter) from an actively growing fungus on a PDA plate. The culture was incubated at 26 ± 2 °C under static conditions in the dark for 3 weeks. Next, 300 μL of veratryl alcohol (Tokyo Chemical Industry Co. Ltd., Tokyo, Japan; 100 mM) in dimethylformamide (Kanto Chemical Co. Inc., Tokyo, Japan) was added to the medium, and oxygen was flushed for 30 s. After two days of treatment, the culture solution was collected using a Miracloth (Calbiochem, San Diego, CA, USA). The filtrate was centrifuged at 9810 × *g* for 10 min at 4 °C. The collected supernatant was transferred into dialysis tubes (27 inches × 32 feet, Sanko Pure Chemical, Ltd., Gifu, Japan) and dehydrated with polyethylene glycol 20,000 (Wako Pure Chemical Co., Osaka, Japan) for 3 h at 7 °C. The concentrated solution in the dialysis tubes was dialyzed overnight against 20 mM succinate buffer (pH 6.5) at 7 °C. The crude enzyme solution was subjected to lignin-degrading enzyme assay. MnP (Paszczynski et al. 1985), LiP (Tien and Kirk 1984), and Lac (Leonowicz and Gzrywnowicz 1981) activities were assayed using vanillidene aceton, veratryl alcohol, and syringaldazine as substrates, respectively. The amount of protein was determined by the Bradford method (Bradford 1976). All enzymes were assayed photometrically in triplicate using a spectrophotometer, and enzymatic activities were expressed as fkat/mg enzyme.

### Manganese peroxidase (MnP)

Manganese peroxidase activity was assayed by the oxidation of vanillylidene acetone (Paszczynski et al. 1985). The reaction was conducted in a 3 mL cuvette containing 1.8 mL of 0.5 M sodium tartrate buffer (pH 5.0), 300 μL of 1 mM MnSO_4_, 300 μL of 1 mM vanillylidene acetone (Sigma Aldrich), 300 μL of appropriate enzyme concentration, and 300 μL of 1 mM H_2_O_2_. The reaction was monitored by the decrease in absorbance at 336 nm (ɛ_336_ = 18,300 M^−1^. cm^−1^) for 2 min. One katal (kat) of MnP activity was defined as the amount of enzyme required to oxidize 1 mol vanillylidene acetone per second.

### Lignin peroxidase (LiP) activity

Lignin peroxidase activity was assayed by the oxidation of veratryl alcohol (Tien and Kirk 1984). The reaction mixture contained 2.91 mL of 0.1 M sodium tartrate buffer (pH 3.0), 30 μL of 100 mM veratryl alcohol (Wako Pure Chemical Industries) aqueous solution, 60 μL of 25 mM H_2_O_2_, and 100 μL of appropriate enzyme concentration in a 3 mL cuvette. The reaction was monitored by increasing the absorbance at 310 nm (ɛ_310_ = 9300 M^−1^. cm^−1^) for 2 min. One katal (kat) of LiP activity was defined as the amount of enzyme required to oxidize 1 mol veratryl alcohol per second.

### Laccase (Lac) activity

Laccase activity was assayed by the oxidation of syringaldazine (Leonowicz and Gzrywnowicz 1981). The reaction was conducted in a 3 mL cuvette containing 750 μL of 0.1 M sodium tartrate buffer (pH 5.3), 200 μL of 0.5 mM syringaldazine (Aldrich Chem. Co), and 1.8 mL an appropriate concentration of enzyme. The reaction was monitored by the increase in absorbance at 525 nm (ɛ_525_ = 65,000 M^−1^. cm^−1^) for 2 min. One katal (kat) of Lac activity was defined as the amount of enzyme required to oxidize 1 mol tetramethoxy-azo-bis-methylenequinone per second.

### Genome sequencing and bioinformatics analysis

For genome sequencing analysis, mycelia of *I*. *obliquus* were cultured in 250 mL of potato dextrose broth (PDB; Becton, Dickinson and Company) medium (24 g/L (w/v)) under agitation at 100 rpm with a shaker (NR-150; TAITEC Corp., Saitama, Japan) for 2 weeks at 25 ± 2 °C in the dark. After culture, the fungal mycelia in PDB medium were collected by filtration through a 0.22 μm-membrane filter (Tullagreen, Corrigtwohill, Co., Cork, Ireland).

Genomic DNA was isolated from fungal mycelia using the cetyltrimethylammonium bromide (CTAB) method (Doyle 1991). Fungal mycelia (0.2 g) were placed in a mortar and ground in liquid nitrogen using a pestle. The powdered mycelia were lysed with 900 μL of CTAB buffer (0.1 M Tris–HCl (pH 9.0), 1.4 M NaCl, and 0.01 M EDTA in 2% CTAB) and incubated at 56 °C for 1 h. The lysed samples were extracted twice with 800 μL of a mixture of chloroform and isoamyl alcohol (Wako Pure Chemical Industries, Ltd.) (24:1, v:v), rotated for 10 min using a microtube rotator MTR-03 (AS ONE International, Inc.), and centrifuged at 6300 × *g* for 10 min at 4 °C using a centrifuge (5418R, Eppendorf, Germany). The aqueous phase was collected and DNA was precipitated with 600 μL of 2-propanol (Wako Pure Chemical Industries, Ltd.). The DNA pellet was collected by centrifugation at 12,300 g for 5 min at 4 °C. The DNA pellet was washed with 400 μL of 70% ethanol and centrifuged at 12,300 × *g* for 5 min at 4 °C. Finally, the DNA pellet was collected and dissolved in 50 μL TE buffer (10 mM Tris–HCl (pH 7.5) and 1 mM EDTA). The dissolved DNA was incubated at 55 °C for 30 min using an Accu Block™ Digital Dry Bath (Labnet International, Inc.). The DNA solution was treated with 1 μL of 10 μg/μL RNase A (QIAGEN, Germany) and incubated at 37 °C for 1 h using an Accu BlockTM Digital Dry Bath (Labnet International, Inc.). The DNA quality was assessed using electrophoresis (Bio-Rad) on 0.7% agarose gel (agarose in TAE buffer containing 2 M Tris–acetate (pH 8.0) and 0.50 mM EDTA), whereas DNA quantity was measured using a Nanodrop spectrophotometer (Thermo Fisher Scientific) and Quantas™ fluorometer (Promega).

Genomic DNA was then fragmented using a Covaris M220 focused ultrasonicator (Covaris, Inc., U.S.A.). Paired-end genome libraries were prepared using the TruSeq Nano DNA library preparation kit (Illumina), according to the manufacturer’s instructions. The quality of the genome libraries was assessed using a Bioanalyzer 2100 (Agilent, U.S.A.) and the quantity was measured using the KAPA library quantification kit (Illumina) according to the manufacturer’s instructions. Genome libraries were then sequenced using a MiSeq sequencer (Illumina) at the Center for Bioscience Research and Education, Utsunomiya University, Japan. The raw sequencing data were deposited at the DNA Data Bank of Japan (DDBJ) as the DDBJ Sequence Read Archive (DRA) under accession no. DRA008573. The raw sequence reads (2 × 301 bp) were trimmed using Trimmomatic ver. 0.36 (Bolger et al. 2014), and reads with low k-mer coverage (< 5) were removed using Khmer ver. 2.0 (Crusoe et al. 2015). Cleaned reads were assembled using SPAdes ver. 3.11.1 (Bankevich et al. 2012). The assembled contig data were deposited at DDBJ as a Bioproject under accession no. PRJDB8445.

The transfer RNA (tRNA) and ribosomal RNA (rRNA) genes of the assembled genome sequence were predicted by performing searches on tRNAscan-SE ver. 1.3.1 (Schattner et al. 2005) and RNAmmer ver. 1.2 (Lagesen et al. 2007), respectively. Specifically, tRNA genes were searched using the “–o” option, and pseudo tRNA genes were eliminated. Protein-coding genes were predicted using the Augustus ver. 3.0.3 (Stanke et al. 2008) with the option ‘–species = phanerochaete_chrysosporium’ and GeneMark-ES (Ter-Hovhannisyan et al. 2008). The predicted protein-coding genes were annotated using several functional annotations based on their homologous sequences. The predicted protein-coding genes were searched using BLAST (Altschul et al. 1990), that is BLASTn and BLASTp. Searches using the BLASTn program against the non-redundant nucleotide database ‘nt’ and the BLASTp program against the protein database ‘Swiss-prot’ were applied with a threshold e-value of 1E^−50^. The predicted protein-coding genes were also mapped onto functional annotations, including Pfam (protein families), GO (Gene Ontology), and KEGG (Kyoto Encyclopedia of Genes and Genomes). InterProScan (Quevillon et al. 2005) was used to predict Pfam and search GO, whereas KEGG was searched using BlastKOALA (Kanehisa et al. 2016).

### cDNA cloning of a putative *mnp* gene

The fungal mycelia were inoculated into Kirk’s medium (Kirk et al. 1986), incubated for 2 weeks, and treated with veratryl alcohol and oxygen for 2 days under the same conditions described in the section on enzyme activity assays. The fungal mycelia were then collected and total RNA was isolated using TRIzol™ reagent (Thermo Fisher Scientific, Waltham, MA, USA). RNA quality was assessed by electrophoresis on 1% agarose gel, and RNA quantity was measured using a Nanodrop spectrophotometer (Thermo Fisher Scientific). One of the 21 putative *mnp* gene from the genome sequence (Gene ID: g1427.t1) was synthesized as cDNA by reverse transcription polymerase chain reaction (RT-PCR). Total RNA was used for 1^st^-strand cDNA synthesis with an oligo (dT) primer and PrimeScript RT Reagent Kit, according to the manufacturer’s guidelines (Takara Bio Inc., Shiga, Japan). cDNA synthesis was performed as follows:15 min reverse transcription reaction at 37 °C, 5 s inactivation reverse transcription at 85 °C, and a final reaction at 4 °C. The synthesized cDNA was then amplified with Ex Taq DNA Polymerase (Takara Bio Inc.) using a pair of specific primers (5′-ATGTCTTTCGTTAAACTCCTTG-3′ and 5′-TTACGGGTCCTTTAGTTTGTC-3′) with the following thermal cycle parameters:1 min initial denaturation at 95 °C, 34 cycles of 30 s denaturation at 95 °C, 30 s annealing at 65 °C, 2 min extension at 72 °C, and 5 min final extension at 12 °C. Pairs of specific primers were designed based on the predicted nucleotide sequence from the genome sequence of *I. obliquus*. The amplified cDNAs were subjected to electrophoresis on 1.5% agarose gel, and the PCR products were purified from the gel using the FastGene® Gel/PCR Extraction Kit (Nippon Genetics Co., Ltd., Tokyo, Japan) and cloned into a pMD20-T vector using the Mighty TA Cloning Kit (Takara Bio Inc.) according to the respective manufacturer’s guidelines. cDNA clones were isolated from bacterial cultures using a HiYield™ Plasmid Mini Kit (RBC Bioscience Corp., Taipei, Taiwan) and sequenced by Macrogen (Seoul, South Korea). Signal peptides were predicted in the deduced amino acid sequence using the signalP-5.0 server. The cDNA clone of putative MnP in *I. obliquus* was then referred to as IoMnP1.

### Structural and phylogenetic analysis of IoMnP1

Multiple alignments of the deduced amino acid sequence of IoMnP1 with other MnPs were prepared using the ClustalX software. The secondary structure of IoMnP1 was predicted using PSIPRED v3.3 on the PSIPRED server (Buchan et al. 2013). The protein structure of IoMnP1 was then modeled using the Swiss-Model automated protein structure homology-modeling server (Biasini et al. 2014) with templates from the Protein Data Bank (PDB) entries 2BOQ, 1MnP, and Lga1 for VP, MnP, and LiP, respectively.

Phylogenetic analysis of the deduced amino acid sequences of 57 Basidiomycete peroxidases was performed using Geneious ver. 9.1 (Kearse et al. 2012). A list of the 57 Basidiomycete peroxidases used in this study is shown in Additional file 1: Table S1. The concatenated amino acid sequences were aligned using MAFFT alignment software ver. 1.4.0 (Katoh et al. 2002). Phylogenetic analysis was conducted using the neighbor-joining method (Saitou and Nei 1987).

## Results

### Lignin-degrading enzyme activities

Table 1 shows that Lac and MnP activities were found in a crude extract from fungal mycelia grown in Kirk’s medium with the addition of veratryl alcohol and oxygen flashing. The activities of Lac and MnP were 0.102 and 0.992 fkt/mg, respectively. The absence of LiP activity indicates that *I. obliquus* is a pathogenic white-rot fungus that does not produce LiP in Kirk’s medium. This fungus most likely expressed only a certain suite of enzymes and metabolites on a single defined artificial medium, and the assays used for the detection of enzymatic activity have limitations.

**Table 1:** Specific activity of lignin-degrading enzymes in *I. obliquus*

Type of enzyme	Specific activity (fkat/mg)
Lac	0.102
MnP	0.992
LiP	0.000

*Lac* laccase, *MnP* manganese peroxidase, *LiP* lignin peroxidase

### Genome features

The statistics of the assembled, predicted, and annotated genomes of *I. obliquus* are summarized in Table 2. The genome of *I. obliquus* was successfully sequenced using next-generation sequencing on the Illumina MiSeq platform and assembled into 15,755 contigs with an N50 length of 9032 bp. The length of the contigs ranged from 200 bp to 199,051 bp with an average length of 2694.8 bp. Overall, the *I. obliquus* genome generated 42.5 Mbp nucleotides with 47.6% GC content. This genome size is within the typical range of genomes in Basidiomycetes ( Floudas et al. 2012; Liu et al. 2012; Olson et al. 2012; Mohanta and Bae 2015; Chen et al. 2016; Li et al. 2018), and somewhat similar to that of *L. edodes* (41.8 Mbp) (Chen et al. 2016). *L. edodes* is a white-rot fungus (order Agaricales in the phylum Basidiomycetes) and is widely known as an edible mushroom owing to its high nutritional and medicinal properties (Chen et al. 2016). Furthermore, the genome assembly of *I. obliquus* contained 2 rRNA genes and 136 tRNA genes (Table 2). These 136 tRNAs corresponded to a full set of 20 amino acids. In total, 21,203 protein-coding genes were identified in this genome assembly. The number of protein-coding genes in the *I. obliquus* genome was within the range of fungi, 11,000–20,000 (Mohanta and Bae 2015).

**Table 2:** Assembly, prediction, and annotation statistics for the *I. obliquus* genome

Characteristic	Statistic
Number of genome contigs	15,755
N50 length (bp)	9032
Shortest contig length (bp)	200
Longest contig length (bp)	199,051
Average contig length (bp)	2694.8
Total number of nucleotides in genomic contig (bp)	42,456,479
GC content (%)	47.6
rRNA genes	2
tRNA genes	136
Number of protein coding genes	21,203
Number of genes annotated by nt database	8280
Number of genes annotated by Swiss-prot database	4188
Number of genes annotated by Pfam	16,190
Number of genes annotated by GO	11,450
Number of genes annotated by KEGG	5277
Number of genes annotated by PHI-base	1621

N50 length, the minimum contig length needed to cover 50% of the genome; *bp* base pair, *GC* guanine-cytosine, *tRNA* transfer RNA, *rRNA* ribosomal RNA

The predicted protein-coding genes were further blasted in the non-redundant nucleotide (nt), Swiss-Prot, Pfam, and KEGG databases. Among the 21,203 predicted protein-coding genes, 8280 and 4188 were significantly similar to those documented in the nt and Swiss-Prot databases, respectively (Table 2). The results of the Pfam search showed that 16,190 genes had structural domains (Table 2 and Additional file 1: Fig. S1). This result revealed that the *I. obliquus* genome is enriched in three conserved domains: the protein kinase domain (PF00069.24; 464 genes), protein tyrosine kinase (PF07714.16; 390 genes), and the AAA ATPase domain (PF13191.5; 388 genes). Moreover, the predicted protein-coding genes were assigned to GO terms to obtain functional information. In total, 11,450 genes were included in the annotation (Table 2). Among these, 7936, 2032, and 1482 genes were mapped to molecular function (MF), biological process (BP), and cellular component (CC), respectively (Additional file 1: Fig. S2).

A total of 5277 genes were documented in the KEGG database, and 257 genes were annotated as “biosynthesis of secondary metabolites” (map01110) (Additional file 1: Fig. S3). The results of KEGG pathway analysis indicated that several pathways involved in the “biosynthesis of secondary metabolites” might be related to the pathways for some medicinal compound biosynthesis reported to date (Saar 1991). In addition, the lanosterol biosynthesis pathway, which is associated with six genes involved in “terpenoid backbone biosynthesis” (map00900; Additional file 1: Fig. S4A) and three genes involved in “steroid biosynthesis” (map00100; Additional file 1: Fig. S4B) was also found in the present study (Additional file 1: Table S2). Meanwhile, the terpenoid backbone biosynthesis pathways seem to be distributed only in the mevalonate (MVA) pathway. A similar result was found in other Basidiomycetes, such as *G. lucidum* (Liu et al. 2012). The putative lanosterol biosynthesis pathway is shown in Additional File 1: Fig. S4C. Lanosterol is a class of chemical compounds that has beneficial properties for human health, such as antitumor effects (Shin and Tamai 2000). In contrast, lanosterol is also considered as an important intermediate in the synthesis of inotodiol and trametenolic acid through hydroxylation and oxidation reactions (Shin and Tamai 2000). These compounds are present in *I. obliquus* and are known to have anti-inflammatory, anticancer, and antitumor effects (Shin and Tamai 2000).

### Genes related to wood degradation

From the functional annotation results, 134 genes were detected in the *I. obliquus* genome, that are potentially involved in the degradation of wood chemical components (Table 3). These genes consisted of 36 candidate cellulase genes, 35 candidate hemicellulase genes, 16 candidate pectinase genes, 37 candidate lignin-modifying enzyme genes, and 10 candidate lignin-degrading auxiliary enzyme genes. Annotation of these genes in nt, Swiss-Prot, Pfam, GO, and KEGG databases is shown in Additional File 2: Table S3. As found in those results, a total of 47 genes were related to lignin degradation and were categorized into two main classes (Table 3; Additional file 2: Table S3). The first contained 37 candidate lignin-modifying enzyme genes consisting of 14 candidate *lac* genes, 21 candidate *mnp* genes, and two candidate dye-decolorizing peroxidase (*DyP*) genes. The second contains 10 candidate lignin-degrading auxiliary enzyme genes, consisting of three candidate glucose oxidase genes, two candidate alcohol oxidase genes, and five candidate aldehyde oxidase genes. The presence of a large variety of enzyme-encoding genes in this fungus may be related to its parasitic nature, where its survival depends on degraded wood chemical components in the host as the primary carbon source of nutrients for growth during colonization, as observed in the pathogenic white-rot fungus *H. irregulare* (Olson et al. 2012; Yakovlev et al. 2013). However, the expression and function of these genes involved in wood degradation identified in this study are still unknown and require more detailed study.

**Table 3:** Candidate genes involved in degradation of wood chemical components by *I. obliquus*

Class	Putative enzyme	Number of genes
Cellulase	Endoglucanase	9
	Exoglucanase	9
	β-Glucanase	15
	Cellobiose dehydrogenase	3
	Total	36
Hemicellulase	β-Xylosidase	6
	Endo-1,4-β-xylanase	7
	4-*O*-Methyl-glucuronoyl methylesterase	1
	Acetylxylan esterase	2
	α-Xylosidase	2
	α-Fucosidase	2
	β-Mannosidase	6
	Endo-β-mannanase	1
	α-Galactosidase	3
	α-L-Arabinofuranosidase	3
	Arabinogalactan endo-β-1,4-galactanase	1
	Feruloyl esterase	1
	Total	35
Pectinase	Pectinesterase	1
	Pectate lyase	4
	Polygalacturonase	1
	Endopolygalacturonase	2
	Exoplygalacturonase	4
	α-L-Rhamnosidase	2
	Rhamnogalacturonan acetylesterase	1
	Arabinan endo-1,5-α-L-arabinosidase	1
	Total	16
Lignin-modifying enzyme	Laccase	14
	Manganese peroxidase	21
	Dye-decolorizing peroxidase	2
	Total	37
Lignin-degrading auxiliary enzyme	Glucose oxidase	3
	Alcohol oxidase	2
	Aldehyde oxidase	5
	Total	10
Total		134

### cDNA cloning of IoMnP1

**Fig. 2:**
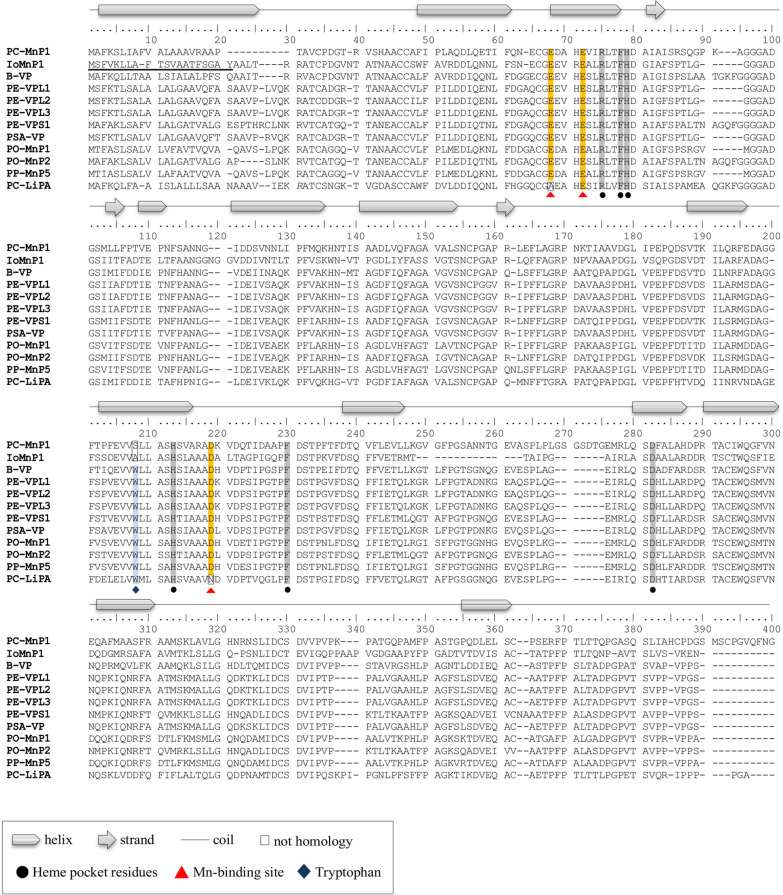
Multiple alignments of the deduced amino acid sequence of IoMnP1. *PC* *Phanerochaete chrysosporium*, *IO* *Inonotus obliquus*, *B* *Bjerkandera* sp. *PE* *Pleurotus eryngii*, *PSA* *P. sapidus*, *PO* *P. ostreatus*, *PP* *P. pulmonarius*, *MnP* manganese peroxidase, *Px* putative manganese peroxidase, *VP* versatile peroxidase, *LiP* lignin peroxidase. The underlined amino acid sequence indicates the signal peptide

In the present study, we selected one of the 21 MnP-encoding genes, which is referred to as *iomnp1*. This is because this gene has the homology with a known *VPs* from *Pleurotus Eryngii* (XP_007269621.1). *iomnp1* was successfully cloned into a vector pMD20-T using the TA cloning method. This gene contains 1,078 nucleotides, encoding 347 deduced amino acids. Additionally, SignalP analysis indicated the presence of a 20-amino acid signal peptide (Fig. 2). These results suggested that IoMnP1 is a typical secreted protein, which is consistent with the fact that it is an extracellular fungal enzyme.

**Fig. 3:**
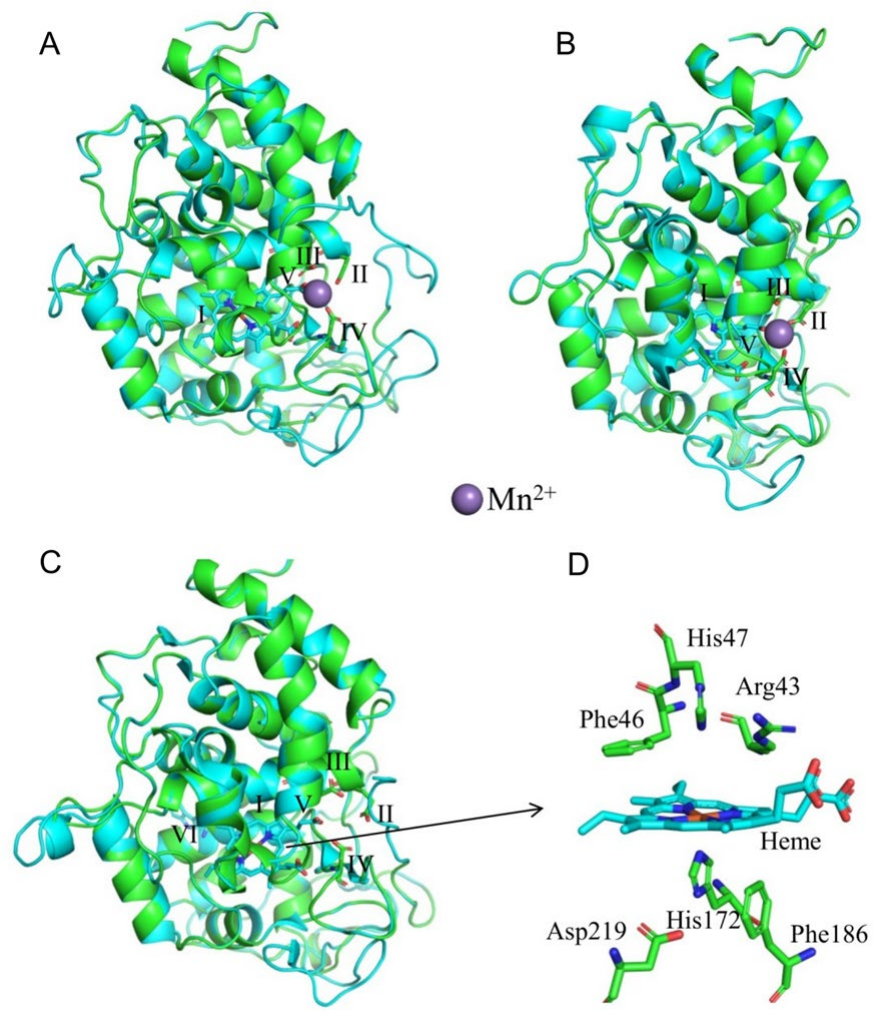
Molecular models and heme environment of IoMnP1. The protein structure of IoMnP1 is represented in templates of the following PDB entries: **A** 1MnP for manganese peroxidase (MnP), **B** 2BOQ for versatile peroxidase (VP), and **C** Lga1 for lignin peroxidase (LiP); **D** heme environment of IoMnP1 from the three templates. I, heme cofactor; II, Glu36; III, Glu40; IV, Asp178; V, internal heme propionate; VI, Trp171 from 1MnP template

A comparison of the deduced amino acid sequence between IoMnP1 and other deduced amino acid sequences of Basidiomycete peroxidases is shown in Fig. 2. The results showed that IoMnP1 has conserved heme pocket residues, that are Arg43, Phe46, His47, His172, Phe189, and Asp219. The heme (prosthetic group) of heme-containing peroxidases is known to be involved in H_2_O_2_ reactions. To investigate the steric orientation of each amino acid and to clarify the catalytic properties of IoMnP1, molecular modeling of the IoMnP1 protein was performed by sequence homology using a model server and built using the templates from PDB entries 1MnP, 2BOQ, and Lga1 for MnP (Fig. 3A), VP (Fig. 3B), and LiP (Fig. 3C), respectively. Figure 3D shows that His47 and His172 were the distal and proximal histidine residues of heme, respectively. Arg43, Phe46, Phe186, and Asp219 were the residues near distal and proximal histidines (distal pocket). Pease et al. (1989) reported that heme-containing peroxidases contain two histidine residues that are essential for their activity (Pease et al. 1989). The proximal histidine residue is predicted to be the axial ligand of heme iron, whereas the distal histidine residue participates in peroxide cleavage (Pease et al. 1989). The results revealed that IoMnP1 contained residues essential for peroxidase activity. In addition, Fig. 2 shows that the deduced heme pocket residues of IoMnP1 are conserved among other genes encoding typical MnP (PC-MnP1), typical VP (B-VP, PE-VPL1, PE-VPL2, PE-VPL3, PE-VPS1, and PSA-VP), putative VP (PO-MnP1, PO-MnP2, and PP-MnP5), and typical LiP (PC-LiPA). These results demonstrate that IoMnP1 is a member of heme-containing peroxidases.

**Fig. 4:**
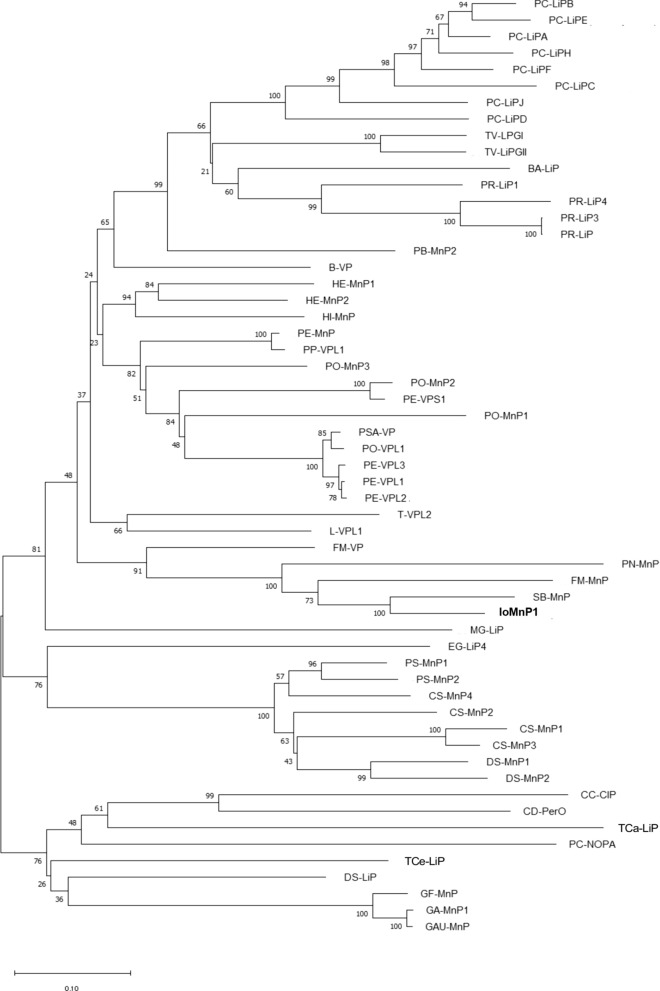
Molecular phylogenetic analysis of 57 genes encoding manganese peroxidases, versatile peroxidases, and lignin peroxidases. *MnP* manganese peroxidase, *VP* versatile peroxidase, *LiP* lignin peroxidase, *Px* putative manganese peroxidase. For other abbreviations refer to Additional file 1: Table S1

In contrast, IoMnP1 also has three acidic residues located in front of the internal heme propionate, which are Glu36, Glu40, and Asp178 (Figs. 3 and 4A, B). These residues are important for the formation of Mn-binding sites ( Ruiz-Dueñas et al. 1999, 2009). As is well known, the Mn-binding site is stabilized by 3 acidic residues (tri-carboxylates of 2 glutamate (Glu) and 1 aspartate (Asp) residues), 1 internal heme propionate, and 2 water molecules in an octahedral molecular geometry (Sundaramoorthy et al. 1994; Ravichandran and Sridhar 2016). At the Mn-binding site, Mn^2+^ is oxidized to Mn^3+^, which is then chelated by organic acids secreted by fungi such as oxalate, glyoxalate, and lactate (Ravichandran and Sridhar 2016). The acidic residues identified in IoMnP1 also showed homology with typical MnP from *P. chrysosporium* (PC-MnP1) and VP from *P. eryngii* (PE-VPL1, PE-VPL2, and PE-VPL3). Therefore, these results indicate that IoMnP1 has catalytic properties analogous to MnP. In contrast, IoMnP1 did not have an exposed tryptophan residue (Trp171 for LiP and Trp164 for VP; Fig. 2). This residue is a characteristic component of LiP and is located on the protein surface (Ruiz-Dueñas et al. 1999, 2009). The absence of this residue in IoMnP1 indicated that the enzyme did not exhibit LiP catalytic activity. In addition, all detected 21 MnPs in the present genome study had no tryptophan residues, indicating that *I. obliquus* has no potential LiPs and VPs. Furthermore, phylogenetic analysis was performed using the 57 deduced amino acid sequences of Basidiomycete peroxidases, including IoMnP1, to investigate the evolutionary relationships between IoMnP1 and other heme-containing peroxidases (Fig. 4). The result showed that IoMnP1 was located in the clade of VP of *Fomitiporia mediterranea* and MnPs from three different species, i.e. *Pyrrhoderma noxium*, *F. mediterranea* and *Sanghuangporus baumii*.

## Discussion

*I. obliquus* is a pathogenic white-rot fungus that parasitizes living trees. During parasitization of host trees, this fungus acquires growth nutrients by degrading chemical components of the wood cell wall. In this study, we investigated the lignin-degrading enzyme activity of *I. obliquus*. Lignin-degrading enzymes assayed were Lac, MnP, and LiP. Based on these results, we identified Lac and MnP activity in *I. obliquus* grown on Kirk medium (Table 1). As only one medium was used to assess enzymatic activity in this study, it is necessary to use other culture media and assay methods in the future.

To clarify these results, we sequenced the genome of *I. obliquus* and investigated genes related to wood degradation. From this genome resource, we found that *mnp* had the highest number of genes encoding lignin-degrading enzymes. The second-highest number of genes in the *I. obliquus* genome was *lac*. MnP and Lac are enzymes that oxidize the phenolic units of lignin. In addition, in plant pathogenic fungi, laccase also plays an important role in detoxification of phenolic compounds involved in plant host defense (Yakovlev et al. 2013) and melanin production (Eisenman and Casadevall 2012). Melanin is a natural pigment produced by *I. obliquus* that protects its cells from sclerotia (Shashkina et al. 2006). Consistent with this enzymatic activity, we did not find any genes that encode LiP. In contrast, genes encoding DyP were found in the *I. obliquus* genome (Table 3; Additional File 2: Table S3). Although the ligninolytic activity of this enzyme is prominent in bacteria (Janusz et al. 2017), it has also been detected in some fungi such as *Termitomyces albuminosus* (Johjima et al. 2003) and *Auricularia auricula-judae* (Liers et al. 2010). In *A. auricula-judae*, DyP is known to degrade nonphenolic lignin units. This is because this enzyme has tyrosine (Tyr337) and tryptophan (Trp377) residues that can participate in LRET, similar to LiP (Liers et al. 2010; Linde et al. 2015). These residues were also present in the deduced amino acid sequences of *DyP* genes in *I. obliquus* (Additional File 1: Fig. S5).

As previously mentioned, heme-containing peroxidases cannot function without the presence of a second class of lignin-degrading enzymes, that is lignin-degrading auxiliary enzymes (Janusz et al. 2017). Genes encoding glucose, alcohol, and aldehyde oxidases were detected in the *I. obliquus* genome. These enzymes provide the H_2_O_2_ necessary for heme-containing peroxidase activity to accomplish the lignin degradation process (Janusz et al. 2017).

Furthermore, we were interested in characterizing *iomnp1*. We successfully cloned this gene into the vector pMD20-T using the TA cloning method (Fig. 2). The deduced amino acid sequence of the *iomnp1* gene was then characterized to investigate its catalytic properties. The results showed that IoMnP1 is a member of the heme-containing peroxidases that show catalytic properties analogous to MnP (Fig. 3). In contrast, the phylogenetic analysis conducted in this study revealed that IoMnP1 was located in the clade of VP of *F. mediterranea* and MnPs from three different species, i.e. *P. noxium*, *F. mediterranea*, and *S. baumii*. In this clade, IoMnP1 and VP of *F. mediterranea* are clearly separated, but it seems to be closely related to that of the MnPs from *P. noxium*, *F. mediterranea*, and *S. baumii* which belong to the same family of Hymenochaetaceae that lack the tryptophan residue (Fig. 4; Additional file 1: Fig. S6). Several studies reported that the presence of Mn-binding residues and the lack of exposed tryptophan residues characterize enzymes as MnPs (Sundaramoorthy et al. 1994, 1997; Yeung et al. 1997; Morgenstern et al. 2010). Moreover, Morgenstern et al. (2010) revealed that the position of the tryptophan residue that characterizes LiPs and VPs is usually occupied by an alanine or serine residue in MnPs. These characteristics are similar to those of IoMnP1 exposed to Mn-binding residues that lack the tryptophan residue whose presence was replaced by alanine residue (Additional file 1: Fig. S6). Therefore, we suggest that IoMnP1 is a member of the MnPs after being confirmed by its cDNA sequence. However, further crystallographic, kinetic, and spectroscopic studies are required to confirm this finding.

## Supplementary Information

**Additional file 1: Fig. S1**. Distribution of *I. obliquus* genes in different Pfam categories. Pfam categories containing more than 100 genes are shown. **Fig. S2**. Distribution of *I. obliquus *genes in different GO terms. GO terms containing more than 100 genes are shown. **Fig. S3**. Distribution of *I. obliquus* genes in different KEGG categories. KEGG categories containing more than 25 genes are shown. **Fig. S4**. Pathways of (A) terpenoid backbone biosynthesis and (B) steroid biosynthesis from the KEGG database. (C) Putative lanosterol biosynthesis pathway in *I. obliquus*. Red EC number, enzyme encoding genes detected in the *I. obliquus *genome. Dashed arrow, enzyme encoding gene not detected. Enzyme encoding genes involved in this pathway are as follows: EC:2.3.1.9, acetyl-CoA acetyltransferase; EC:2.3.3.10, hydroxymethylglutaryl-CoA synthase A; EC:1.1.1.34, 3-hydroxy-3-methylglutaryl-coenzyme A reductase; EC:2.7.4.2, phosphomevalonate kinase; EC: 4.1.1.33, diphosphomevalonate decarboxylase; EC:2.5.1.10, farnesyl pyrophosphate synthase; EC: 2.5.1.21, squalene synthase; EC: 1.14.1417, squalene epoxidase; EC: 5.4.99.7, lanosterol synthase. **Fig. S5**. Multiple alignment of the deduced amino acid sequence of DyPs. AA-DyP, DyP of *Auricularia auricula-judae* (Accession No. 4W7L_A); IO-DyP.1 and IO-DyP.2, DyPs of *Inonotus obliquus* with gene IDs. MSTRG.14052.1 and g3844.t1, respectively; SB-DyP, DyP of *Sanghuangporus baumii* (Accession No. OCB85293.1); HI-DyP, DyP of *Heterobasidion irregulare* (Accession No. XP_009544629.1). **Fig. S6**. Multiple alignments of the deduced amino acid sequence of IoMnP1. PO, *Pleurotus ostreatus*; PN, *Pyrrhoderma noxium*; FM, *Fomitiporia mediterranea*; SB, *Sanghuangporus baumii*; IO, *Inonotus obliquus*; B, *Bjerkandera* sp.; PE, *P. eryngii*; PC, *Phanerochaete chrysosporium*; TCe, *Triuncina cervine*; MnP, manganese peroxidase; Px, putative versatile peroxidase; VP, versatile peroxidase; LiP, lignin peroxidase. The underlined amino acid sequence indicates the signal peptide. **Table S1. **List of the 57 Basidiomycete peroxidases used in this study. **Table S2**. Enzyme encoding genes involved in lanosterol biosynthesis based on KEGG annotation.

**Additional file 2: Table. S3**. Annotation of genes related to wood degradation.

## Data Availability

The raw sequencing data were deposited at DNA Data Bank of Japan (DDBJ) as a DDBJ Sequence Read Archive (DRA) under the accession no. DRA008573, and the assembled contig data were deposited at DDBJ as a Bioproject under the accession no. PRJDB8445.

## Abbreviations

Lac: Laccase
MnP: Manganese peroxidase
LiP: And lignin peroxidase
VP: Versatile peroxidase
*I. obliquus*: *Inonotus obliquus* Strain IO-B2 (NBRC 113408)
LRET: Long-range electron transfer
cDNA: Complementary DNA
NBRC: NITE Biological Resource Center
PDA: Potato-dextrose-agar
PDB: Potato-dextrose-broth
tRNA: Transfer RNA
rRNA: And ribosomal RNA
BLAST: Basic Local Alignment Search Tool
BLASTn: Nucleotide BLAST
BLASTp: Protein BLAST
Pfam: Protein families
GO: Gene ontology
KEGG: Kyoto encyclopedia of genes and genomes
RT-PCR: Reverse transcription polymerase chain reaction
PCR: Polymerase chain reaction
IoMnP1: *Inonotus obliquus**—*Putative manganese peroxidase
PDB: Protein data bank
MF: Molecular function
BP: Biological process
CC: Cellular component
*G. lucidum*: *Ganoderma lucidum*
*L. edodes*: *Lentinula edodes*
DyP: Dye-decolorizing peroxidase
*H. irregulare*: *Heterobasidion irregular*
Arg: Arginine residue
Phe: Phenylalanine residue
His: Histidine residue
Asp: Aspartic acid residue
Glu: Glutamic acid residue
*Trp*: *tryptophan residue*
*P. chrysosporium*: *Phanerochaete* *chrysosporium*
*P. eryngii*: Pleurotus eryngii
*A. auricula-judae*: *Auricularia auricula-judae*
*P. noxium*: *Pyrrhoderma noxium*
*F. mediterranea*: *Fomitiporia mediterrane*
*S. baumii*: *Sanghuangporus baumii*
