# Supplementary material for: Lignin-degrading enzymes from a pathogenic canker-rot fungus Inonotus obliquus strain IO-B2
Source: AMB Express. 2023 Jun 11;13:59. doi: 10.1186/s13568-023-01566-3 (PMC10258189; doi:10.1186/s13568-023-01566-3)
Supplement: Supplementary file 1 — Additional file 1: Fig. S1. Distribution of I. obliquus genes in different Pfam categories. Pfam categories containing more than 100 genes are shown. Fig. S2. Distribution of I. obliquus genes in different GO terms. GO terms containing more than 100 genes are shown. Fig. S3. Distribution of I. obliquus genes in different KEGG categories. KEGG categories containing more than 25 genes are shown. Fig. S4. Pathways of (A) terpenoid backbone biosynthesis and (B) steroid biosynthesis from the KEGG database. (C) Putative lanosterol biosynthesis pathway in I. obliquus. Red EC number, enzyme encoding genes detected in the I. obliquus genome. Dashed arrow, enzyme encoding gene not detected. Enzyme encoding genes involved in this pathway are as follows: EC:2.3.1.9, acetyl-CoA acetyltransferase; EC:2.3.3.10, hydroxymethylglutaryl-CoA synthase A; EC:1.1.1.34, 3-hydroxy-3-methylglutaryl-coenzyme A reductase; EC:2.7.4.2, phosphomevalonate kinase; EC: 4.1.1.33, diphosphomevalonate decarboxylase; EC:2.5.1.10, farnesyl pyrophosphate synthase; EC: 2.5.1.21, squalene synthase; EC: 1.14.1417, squalene epoxidase; EC: 5.4.99.7, lanosterol synthase. Fig. S5. Multiple alignment of the deduced amino acid sequence of DyPs. AA-DyP, DyP of Auricularia auricula-judae (Accession No. 4W7L_A); IO-DyP.1 and IO-DyP.2, DyPs of Inonotus obliquus with gene IDs. MSTRG.14052.1 and g3844.t1, respectively; SB-DyP, DyP of Sanghuangporus baumii (Accession No. OCB85293.1); HI-DyP, DyP of Heterobasidion irregulare (Accession No. XP_009544629.1). Fig. S6. Multiple alignments of the deduced amino acid sequence of IoMnP1. PO, Pleurotus ostreatus; PN, Pyrrhoderma noxium; FM, Fomitiporia mediterranea; SB, Sanghuangporus baumii; IO, Inonotus obliquus; B, Bjerkandera sp.; PE, P. eryngii; PC, Phanerochaete chrysosporium; TCe, Triuncina cervine; MnP, manganese peroxidase; Px, putative versatile peroxidase; VP, versatile peroxidase; LiP, lignin peroxidase. The underlined amino acid sequence indicates the signal peptide. [file 13568_2023_1566_MOESM1_ESM.docx]

**Additional file 1**

**
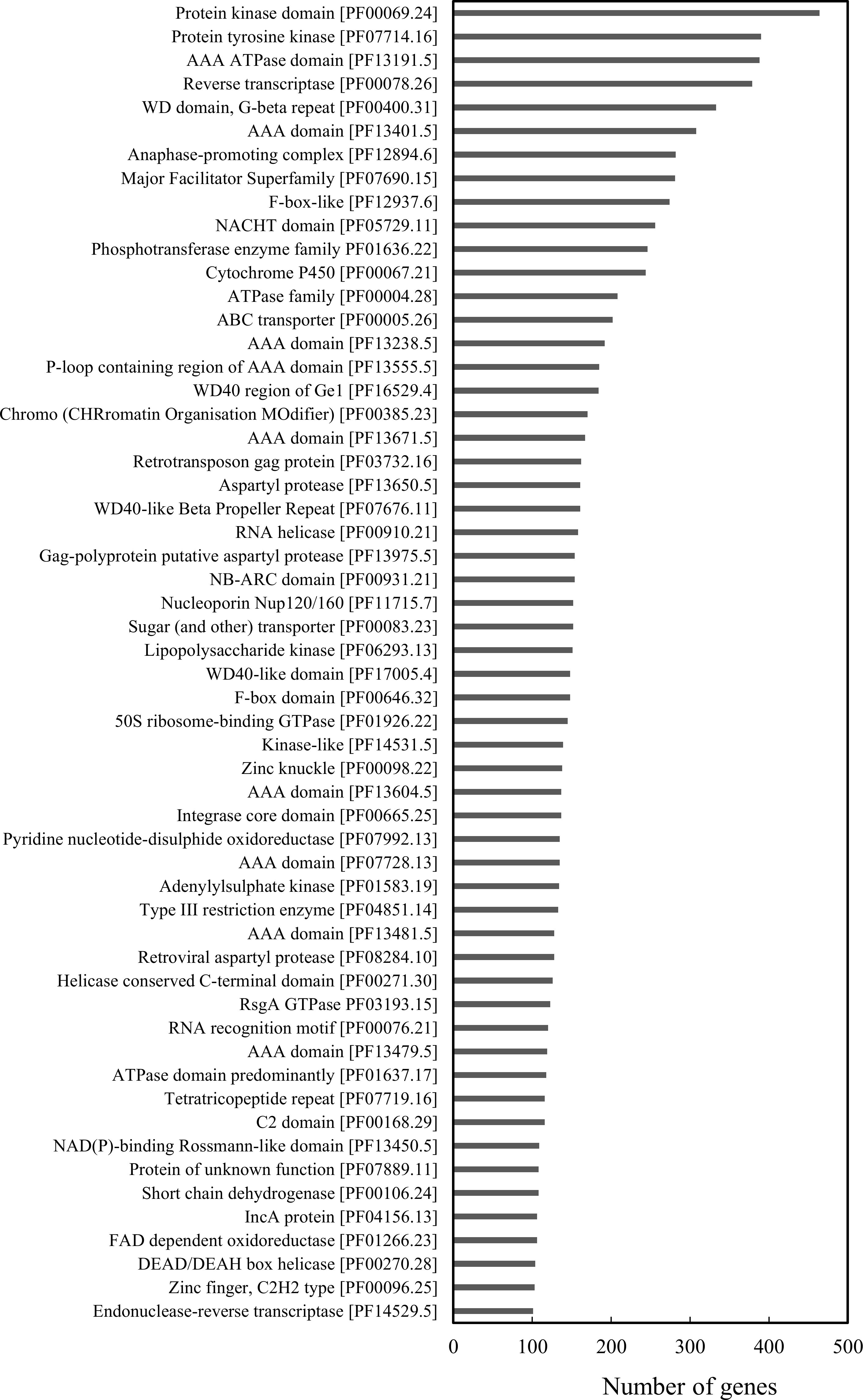
**

**Fig. S1.** Distribution of *I. obliquus* genes in different Pfam categories. Pfam categories containing more than 100 genes are shown.

**
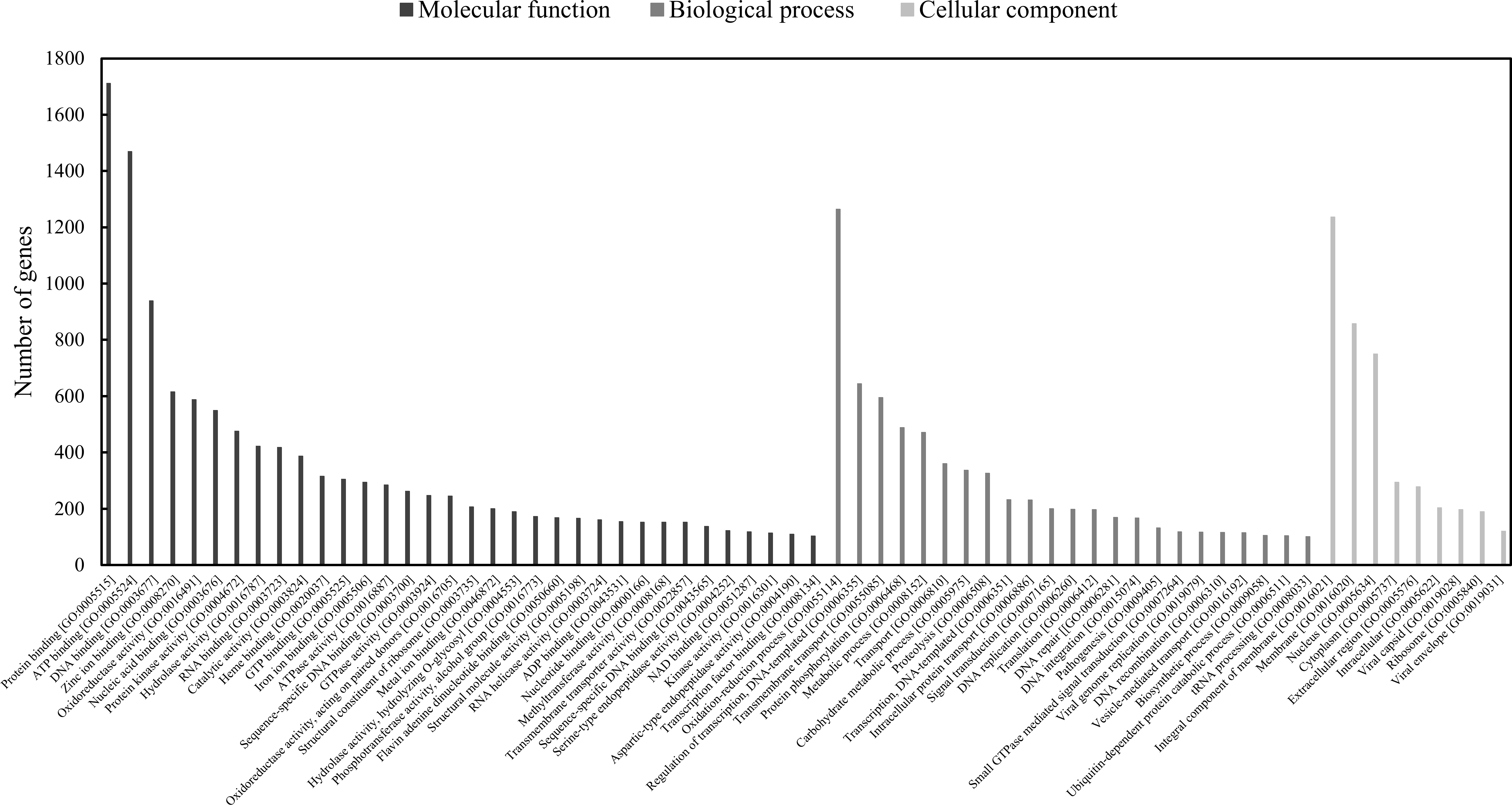
**

**Fig. S2.** Distribution of *I. obliquus* genes in different GO terms. GO terms containing more than 100 genes are shown.


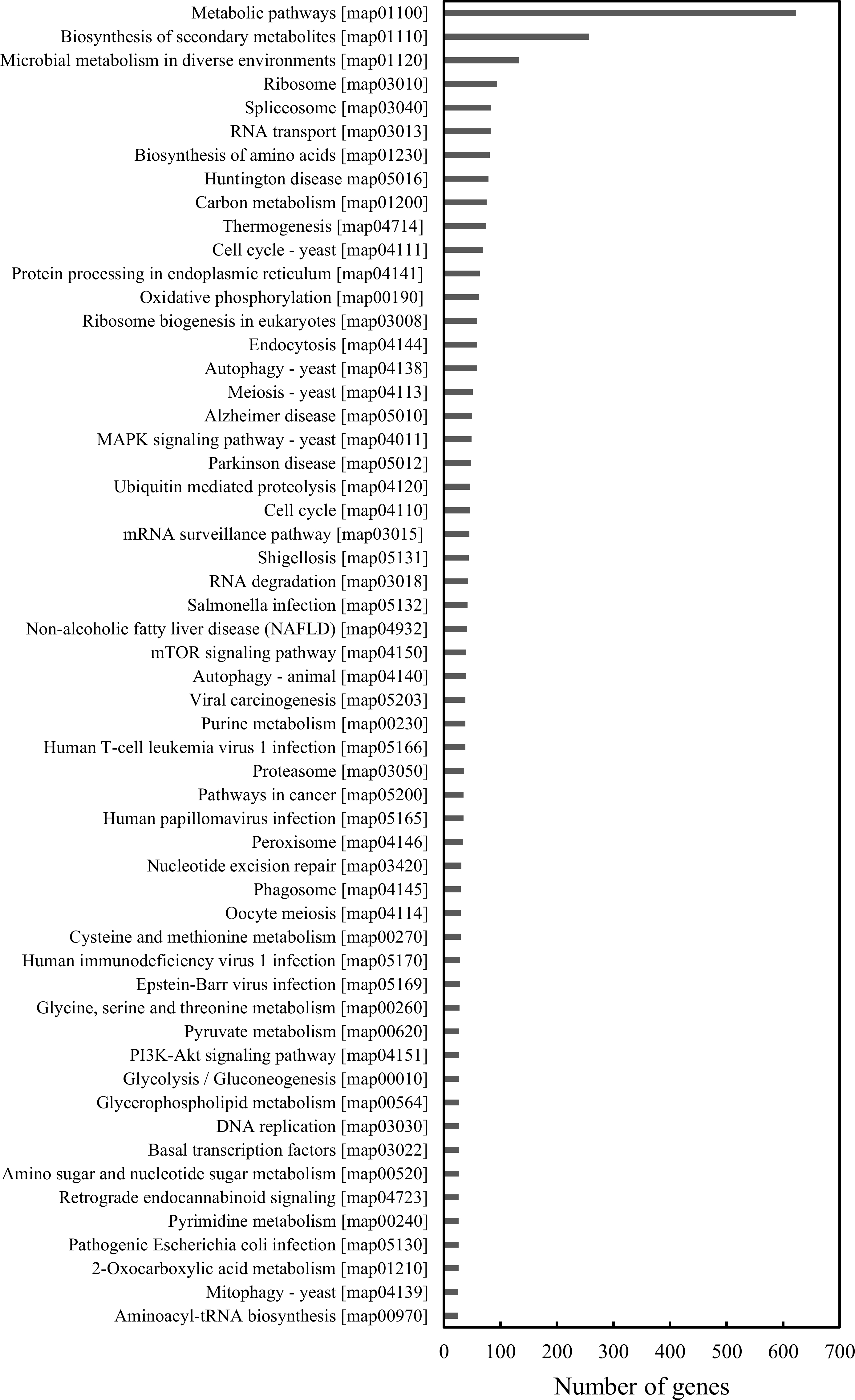


**Fig. S3.** Distribution of *I. obliquus* genes in different KEGG categories. KEGG categories containing more than 25 genes are shown.

| 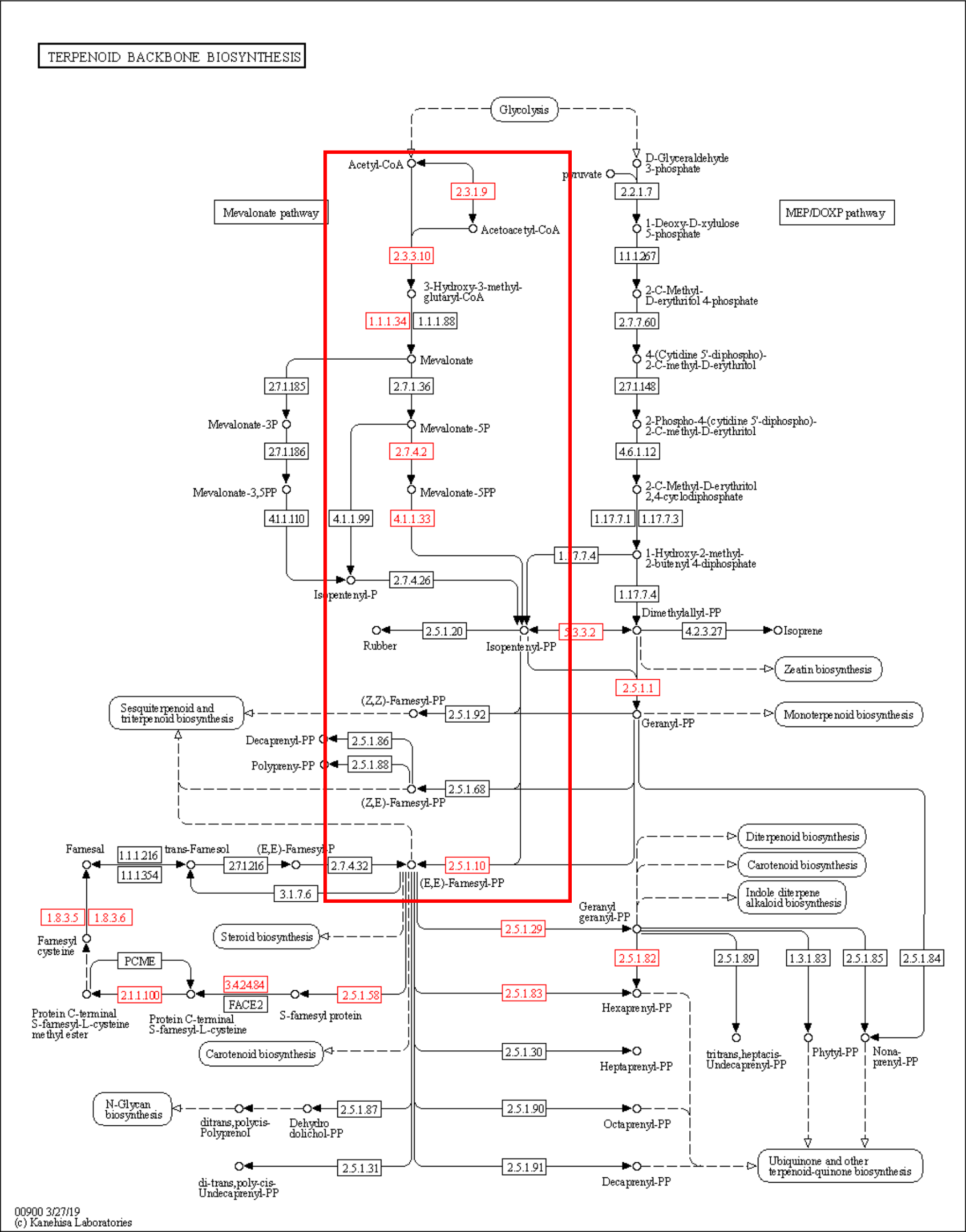  A | **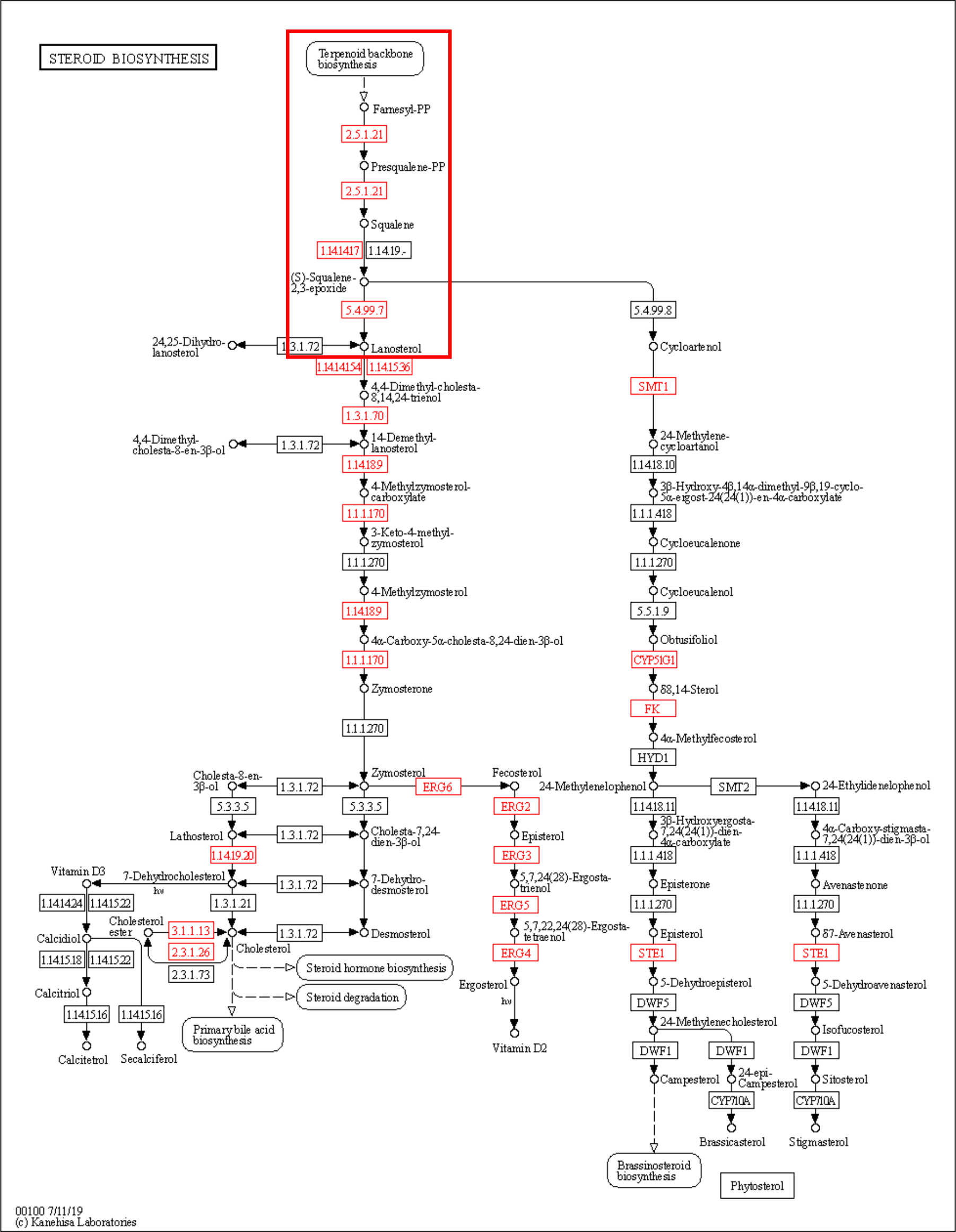**  B |
| --- | --- |
| **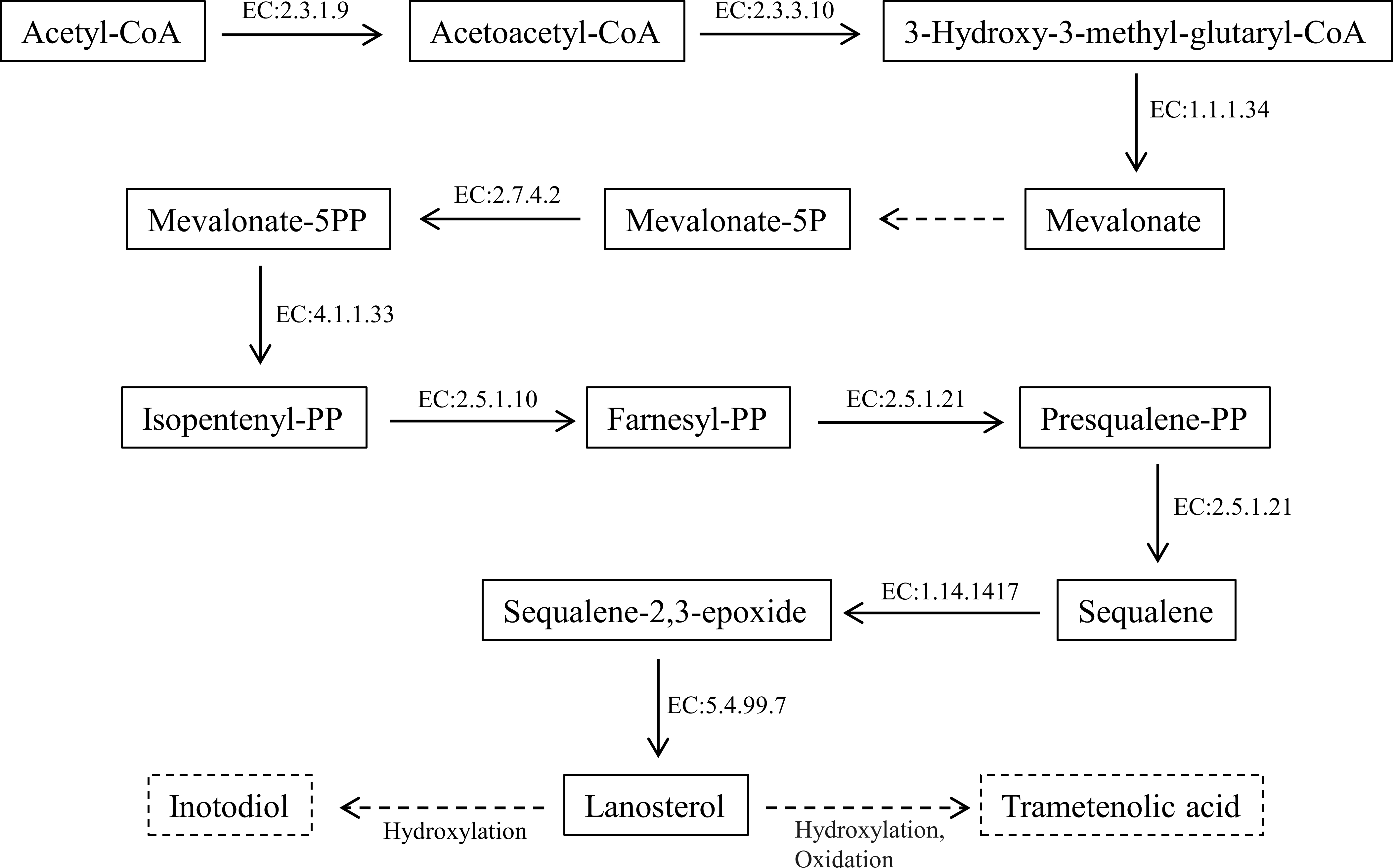**  C | |

**Fig. S4.** Pathways of (A) terpenoid backbone biosynthesis and (B) steroid biosynthesis from the KEGG database. (C) Putative lanosterol biosynthesis pathway in *I. obliquus*. Red EC number, enzyme encoding genes detected in the *I. obliquus* genome. Dashed arrow, enzyme encoding gene not detected. Enzyme encoding genes involved in this pathway are as follows: EC:2.3.1.9, acetyl-CoA acetyltransferase; EC:2.3.3.10, hydroxymethylglutaryl-CoA synthase A; EC:1.1.1.34, 3-hydroxy-3-methylglutaryl-coenzyme A reductase; EC:2.7.4.2, phosphomevalonate kinase; EC: 4.1.1.33, diphosphomevalonate decarboxylase; EC:2.5.1.10, farnesyl pyrophosphate synthase; EC: 2.5.1.21, squalene synthase; EC: 1.14.1417, squalene epoxidase; EC: 5.4.99.7, lanosterol synthase.


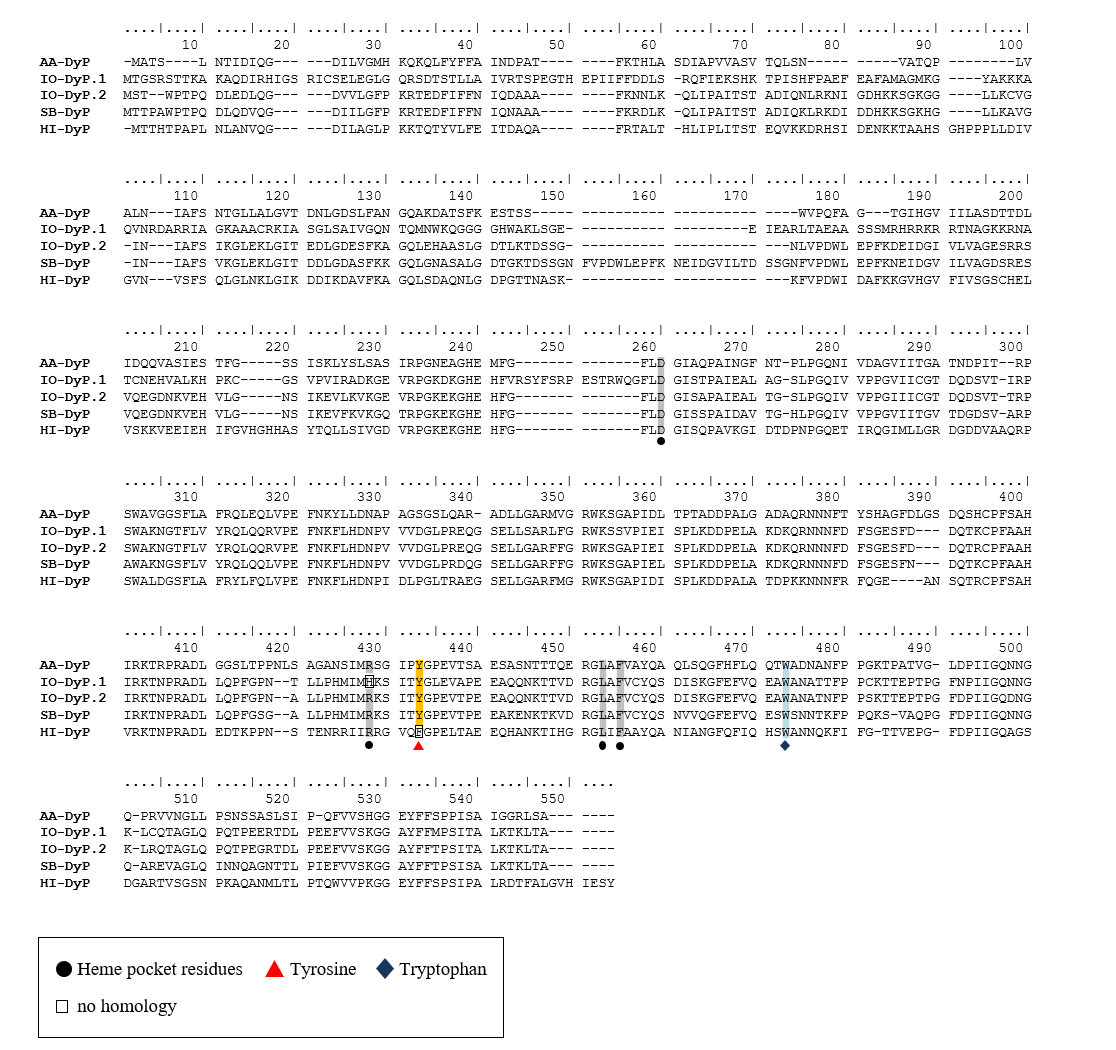


**Fig. S5.** Multiple alignment of the deduced amino acid sequence of DyPs. AA-DyP, DyP of *Auricularia auricula-judae* (Accession No. 4W7L_A); IO-DyP.1 and IO-DyP.2, DyPs of *Inonotus obliquus* with gene IDs. MSTRG.14052.1 and g3844.t1, respectively; SB-DyP, DyP of *Sanghuangporus baumii* (Accession No. OCB85293.1); HI-DyP, DyP of *Heterobasidion irregulare* (Accession No. XP_009544629.1).


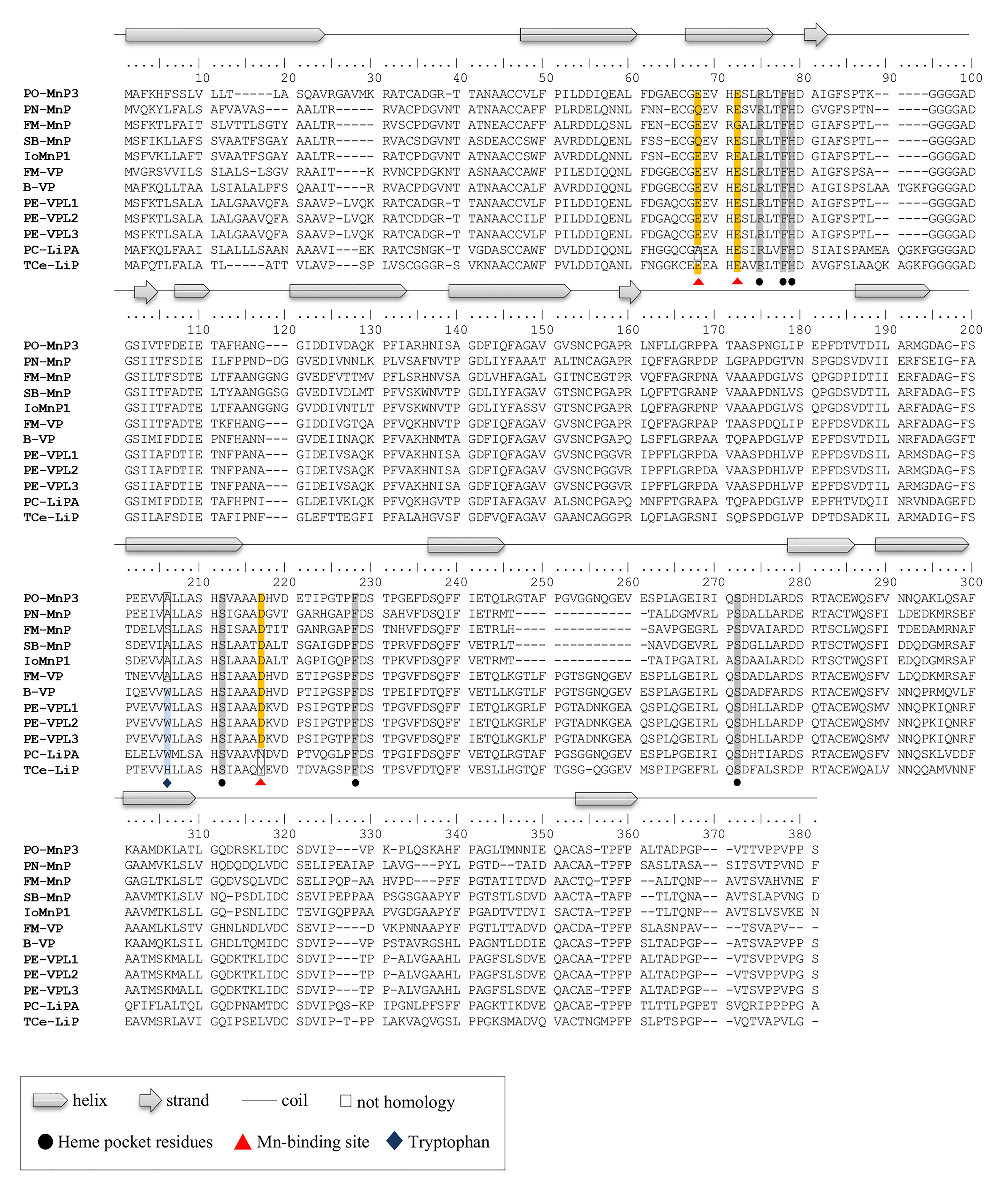


**Fig. S6.** Multiple alignments of the deduced amino acid sequence of IoMnP1. PO, *Pleurotus ostreatus*; PN, *Pyrrhoderma noxium*; FM, *Fomitiporia mediterranea*; SB, *Sanghuangporus baumii*; IO, *Inonotus obliquus*; B, *Bjerkandera* sp.; PE, *P. eryngii*; PC, *Phanerochaete chrysosporium*; TCe, *Triuncina cervine*; MnP, manganese peroxidase; Px, putative versatile peroxidase; VP, versatile peroxidase; LiP, lignin peroxidase. The underlined amino acid sequence indicates the signal peptide.

**Table S1.** List of the 57 basidiomycete peroxidases used in this study.

| Name | Species | Putative enzyme | Acc. No.^a^ |
| --- | --- | --- | --- |
| B-VP | *Bjerkandera* sp. | Typical VP | AAO47909 |
| BA-LiP | *Bjerkandera adusta* | Typical LiP | 1906181A |
| CD-PerO | *Coprinellus disseminatus* | Peroxidase | AAZ14938 |
| CC-CIP | *Coprinopsis cinerea* | Coprinopsis cinerea peroxidase | CAA50060 |
| CS-MnP1 | *Ceriporiopsis subvermispora* | Typical MnP | AAB03480 |
| CS-MnP2 |  | Typical MnP | AAD43581 |
| CS-MnP3 |  | Typical MnP | AAD45725 |
| CS-MnP4 |  | Typical MnP | AAO61784 |
| DS-MnP1 | *Dichomitus squalens* | Typical MnP | AAF31330 |
| DS-MnP2 |  | Typical MnP | AAF31330 |
| DS-LiP |  | Typical LiP | EJF59849 |
| EG-LiP4 | *Exidia glandulosa* | Typical LiP | KZV82075 |
| FM-MnP | *Fomitiporia mediterranea* | Typical MnP | XP_007261871 |
| FM-VP |  | Typical VP | XP_007269621 |
| GA-MnP1 | *Ganoderma applanatum* | Putative LiP | BAA88392 |
| GAU-MnP | *Ganoderma austral* | Putative LiP | AAB77244 |
| GF-MnP | *Goniophlebium formosanum* | Putative LiP | ABB77243 |
| HE-MnP1 | *Hericium erinaceus* | Typical MnP | ADK26471 |
| HE-MnP2 |  | Typical MnP | AFD50190 |
| HI-MnP | *Heterobasidion irregulare* | Typical MnP | XP_009553237 |
| IoMnP1 | *Inonotus obliquus* | Putative MnP | Present study |
| L-VPL1 | *Leucoagaricus sp. SymC.cos* |  | KXN90156 |
| MG-LiP | *Mycena galopus* | Typical LiP | KAF8194461 |
| PB-MnP2 | *Polyporus brumalis* | Typical MnP | RDX44186 |
| PC-LiPA | *Phanerochaete chrysosporium* | Typical LiP | AAA53109 |

Note: ^a^, amino acid sequence accession numbers from the NCBI database; MnP, manganese peroxidase; LiP, lignin peroxidase; VP, versatile peroxidase; Px, putative manganese peroxidase; putative VP, unclassified or described as LiP and MnP; putative LiP, previously described as MnP.

**Table S1.** Continued.

| Name | Species | Putative enzyme | Acc. No.^a^ |
| --- | --- | --- | --- |
| PC-LiPB | *Phanerochaete chrysosporium* | Typical LiP | AAA33741 |
| PC-LiPC |  | Typical LiP | AAA33739 |
| PC-LiPD |  | Typical LiP | CAA33621 |
| PC-LiPE |  | Typical LiP | AAA33738 |
| PC-LiPF |  | Typical LiP | AAA33736 |
| PC-LiPH |  | Typical LiP | AAA56852 |
| PC-LiPJ |  | Typical LiP | AAD46494 |
| PC-NOPA |  | Peroxidase | AAU82081 |
| PE-MnP | *Pleurotus eryngii* | Typical MnP | KAF9497322 |
| PE-VPL1 |  | Typical VP | AAD01401 |
| PE-VPL2 |  | Typical VP | AAD01404 |
| PE-VPL3 |  | Typical VP | CAD56164 |
| PE-VPS1 |  | Typical VP | AAD54310 |
| PN-MnP | *Pyrrhoderma noxium* | Typical MnP | PAV17819 |
| PO-MnP1 | *Pleurotus ostreatus* | Putative VP | AAA84396 |
| PO-MnP2 |  | Putative VP | CAB51617 |
| PO-MnP3 |  | Other MnP | BAA33449 |
| PO-VPL1 |  | Typical VP | XP_036631932 |
| PP-VPL1 | *Pleurotus pulmonarius* | Typical VP | KAF4567729 |
| PR-LiP | *Phlebia radiata* | Typical LiP | AAW71986 |
| PR-LiP1 |  | Typical LiP | AAW59419 |
| PR-LiP3 |  | Typical LiP | P20010 |
| PR-LiP4 |  | Typical LiP | AAW66483 |
| PS-MnP1 | *Phanerochaete sordida* | Other MnP | BAC06185 |
| PS-MnP2 |  | Typical MnP | BAC06186 |
| PSA-VP | *Pleurotus sapidus* | Putative VP | CAJ01576 |
| SB-MnP | *Sanghuangporus baumii* | Typical MnP | OCB89183 |
| T-VPL2 | *Termitomyces sp.* |  | AG5726941 |
| TCa-LiP | *Taiwanofungus camphoratus* | Typical LiP | ACA48489 |
| TCe-LiP | *Triuncina cervina* | LiP (Tyr) | AB191466 |
| TV-LiPG1 | *Trametes versicolor* | Typical LiP | AAA34049 |
| TV-LiPGII |  | Typical LiP | CAA53333 |

Note: ^a^, amino acid sequence accession numbers from the NCBI database; MnP, manganese peroxidase; LiP, lignin peroxidase; VP, versatile peroxidase; Px, putative versatile peroxidase; putative VP, unclassified or described as LiP and MnP; putative LiP, previously described as MnP

**Table S2.** Enzyme encoding genes involved in lanosterol biosynthesis based on KEGG annotation.

| Map ID | Pathway annotation | EC number | KEGG ID | Putative enzyme |
| --- | --- | --- | --- | --- |
| map00900 | Terpenoid backbone biosynthesis | 2.3.1.9 | K00626 | Acetyl-CoA C-acetyltransferase |
|  |  | 2.3.3.10 | K01641 | Hydroxymethylglutaryl-CoA synthase |
|  |  | 1.1.1.34 | K00021 | Hydroxymethylglutaryl-CoA reductase (NADPH) |
|  |  | 2.7.4.2 | K00938 | Phosphomevalonate kinase |
|  |  | 4.1.1.33 | K01597 | Diphosphomevalonate decarboxylase |
|  |  | 2.5.1.10 | K00787 | Farnesyl diphosphate synthase |
| map00100 | Steroid biosynthesis | 2.5.1.21 | K00801 | Farnesyl-diphosphate farnesyltransferase |
|  |  | 1.14.14.17 | K00511 | Squalene monooxygenase |
|  |  | 5.4.99.7 | K01852 | Lanosterol synthase |

Note: EC number, enzyme commission number; KEGG ID, accession number from the KEGG annotation.
